# Anti-Hyperglycemic Effects of Alpha-Mangostin in Animal Models: A Systematic Review and Meta-Analysis

**DOI:** 10.3390/life16060906

**Published:** 2026-05-28

**Authors:** Moragot Chatatikun, Fumitaka Kawakami, Motoki Imai, Ratana Netphakdee, Aman Tedasen, Jongkonnee Thanasai, Wiyada Kwanhian Klangbud, Atthaphong Phongphithakchai

**Affiliations:** 1 School of Allied Health Sciences, Walailak University, Nakhon Si Thammarat 80160, Thailand; moragot.ch@wu.ac.th (M.C.); ratana.ne@mail.wu.ac.th (R.N.); aman.te@wu.ac.th (A.T.); 2 Research Excellence Center for Innovation and Health Products (RECIHP), Walailak University, Nakhon Si Thammarat 80160, Thailand; 3 Department of Regulation Biochemistry, Graduate School of Medical Sciences, Kitasato University, Sagamihara 252-0373, Japan; kawakami@kitasato-u.ac.jp; 4 Department of Health Administration, School of Allied Health Sciences, Kitasato University, Sagamihara 252-0373, Japan; 5 Regenerative Medicine and Cell Design Research Facility, School of Allied Health Science, Kitasato University, Sagamihara 252-0373, Japan; imai-m@kitasato-u.ac.jp; 6 Department of Molecular Diagnostics, School of Allied Health Sciences, Kitasato University, Sagamihara 252-0373, Japan; 7 Department of Applied Tumor Pathology, Graduate School of Medical Sciences, Kitasato University, Sagamihara 252-0373, Japan; 8 Faculty of Medicine, Mahasarakham University, Mahasarakham 44000, Thailand; jongkonnee@msu.ac.th; 9 Medical Technology Program, Faculty of Science, Nakhon Phanom University, Nakhon Phanom 48000, Thailand; wiyadakwanhian@gmail.com; 10 Nephrology Unit, Division of Internal Medicine, Faculty of Medicine, Prince of Songkla University, Songkhla 90110, Thailand

**Keywords:** alpha-mangostin, diabetes mellitus, hyperglycemia, glucose, glycated hemoglobin, insulin

## Abstract

Diabetes mellitus, particularly type 2 diabetes mellitus, is a growing global health burden characterized by chronic hyperglycemia and insulin resistance. Alpha-mangostin (AM), a xanthone from *Garcinia mangostana* pericarp, exhibits antioxidant, anti-inflammatory, and metabolic regulatory properties in preclinical models. We performed a systematic review and meta-analysis of controlled in vivo studies to assess AM’s anti-hyperglycemic effects. Fourteen studies (25 comparisons) in rodent models of diabetes or hyperglycemia were included. Primary outcome was blood glucose; secondary outcomes were glycated hemoglobin (HbA1c), insulin, and homeostatic model assessment of insulin resistance (HOMA-IR). Random-effects meta-analysis demonstrated that AM significantly reduced fasting blood glucose (mean difference (MD) = −8.75 mmol/L; 95% CI: −10.73 to −6.78; *p* < 0.001) and HbA1c (MD = −2.20%; 95% CI: −3.07 to −1.32; *p* < 0.001). AM did not significantly alter circulating insulin (Hedges’ g = 0.43; 95% CI: −0.62 to 1.49; *p* = 0.42) but improved insulin resistance as measured by HOMA-IR (MD = −0.90; 95% CI: −1.68 to −0.12; *p* = 0.02). Subgroup, sensitivity, and risk-of-bias analyses supported the robustness of the glucose-lowering association, although substantial between-study heterogeneity was present. Overall, this study provides preclinical evidence that AM exerts significant antihyperglycemic and insulin-sensitizing effects, supporting its potential as a multitarget metabolic modulator. Further standardized and mechanistically informed studies are warranted to facilitate translational progression.

## 1. Introduction

Diabetes mellitus is a major global public health problem. Prevalence is similar in men and women, peaks at ages 75–79, is higher in urban (12.1%) than rural (8.3%) areas, and greater in high-income (11.1%) versus low-income (5.5%) countries. The largest relative increase by 2045 is expected in middle-income countries (21.1%) [1]. Type 2 diabetes mellitus (T2DM) is a progressive metabolic disorder driven by chronic insulin resistance, impaired β-cell function, and persistent hyperglycemia, contributing to rising global morbidity [2]. T2DM typically arises later in life and is commonly linked to obesity, physical inactivity, or consumption of a high-calorie diet [3]. Current therapies include biguanides, sulfonylureas, meglitinides, thiazolidinediones (TZDs), dipeptidyl-peptidase-4 (DPP-4) inhibitors, sodium–glucose cotransporter-2 (SGLT2) inhibitors, and glucagon-like peptide-1 (GLP-1) receptor agonists, as outlined in the American Diabetes Association (ADA) treatment guide [4,5]. These medications act through diverse mechanisms, including reducing hepatic glucose output, stimulating pancreatic insulin secretion, enhancing peripheral insulin sensitivity, increasing incretin activity, or promoting renal glucose excretion [4,5]. Their use is often limited by adverse effects such as gastrointestinal intolerance with metformin, hypoglycemia and weight gain with sulfonylureas and meglitinides, edema and fracture risk with thiazolidinediones, upper-respiratory symptoms with DPP-4 inhibitors, and genital infections or volume depletion with SGLT2 inhibitors [6,7,8,9,10]. Although GLP-1 receptor agonists provide strong glycemic, cardiovascular, and weight-loss benefits, their use is constrained by gastrointestinal side effects, gallbladder disease risk, rare pancreatitis, and high cost [11]. In addition to GLP-1 receptor agonists, emerging dual incretin receptor agonists, such as tirzepatide, have demonstrated enhanced glycemic and metabolic benefits through combined glucose-dependent insulinotropic polypeptide (GIP) and GLP-1 receptor activation [12]. These limitations highlight the need for safer multitarget agents and have motivated interest in natural bioactive compounds.

Alpha-mangostin (AM), a major prenylated xanthone isolated from the pericarp of *Garcinia mangostana*, has emerged as a bioactive compound with metabolic effects. Preclinical studies consistently demonstrate that AM exerts strong antioxidant and anti-inflammatory actions, largely through suppression of reactive oxygen species generation and inhibition of nuclear factor-κB (NF-κB) signaling, while activating cytoprotective pathways such as nuclear factor erythroid 2–related factor 2 (Nrf2)/heme oxygenase-1 (HO-1) [13,14,15,16]. At the metabolic level, AM modulates key regulators of lipid and glucose metabolism, including downregulation of sterol regulatory element-binding protein-1 (SREBP-1), fatty acid synthase (FAS), stearoyl-CoA desaturase-1 (SCD-1), and acetyl-CoA carboxylase (ACC), thereby reducing lipogenesis and ectopic lipid accumulation [17,18,19]. These effects are mechanistically relevant, as lipotoxicity and oxidative stress are central contributors to insulin resistance and β-cell dysfunction in T2DM. Previous evidence indicates that AM improves glucose homeostasis primarily by enhancing insulin sensitivity rather than stimulating insulin secretion. Mechanistically, AM modulates cellular energy metabolism by activating 5′-adenosine monophosphate–activated protein kinase (AMPK) and sirtuin 1 (SIRT1) signaling pathways, upregulating peroxisome proliferator-activated receptor gamma (PPARγ), and facilitating glucose transporter (GLUT) translocation, thereby enhancing peripheral glucose uptake and suppressing hepatic gluconeogenesis [18,19,20]. In diabetic cardiometabolic and endothelial models, AM attenuates protein kinase B (AKT)/forkhead box protein O1 (FOXO1)/cluster of differentiation 36 (CD36)–mediated lipid influx, reduces oxidative injury, and restores insulin signaling efficiency. [17,21]. Additionally, AM mitigates endoplasmic reticulum stress and ceramide accumulation by inhibiting acid sphingomyelinase activation, further improving insulin sensitivity and endothelial function [21,22]. These converging molecular actions position AM as a multitarget metabolic regulator capable of addressing the intertwined mechanisms of hyperglycemia, insulin resistance, inflammation, and oxidative stress.

To date, no comprehensive synthesis has quantitatively evaluated the totality of preclinical evidence regarding the glucose-lowering efficacy of AM. Therefore, the present study aimed to systematically review and meta-analyze available controlled animal studies investigating the effects of isolated or purified AM on glycemic outcomes, including blood glucose, glycated hemoglobin, insulin levels, and insulin resistance indices. By integrating results across diverse experimental settings and exploring sources of heterogeneity through subgroup and sensitivity analyses, this work seeks to clarify the preclinical evidence base and inform future mechanistic and translational research on AM as a potential multitarget antihyperglycemic agent.

## 2. Materials and Methods

### 2.1. Protocol Registration and PICOS Criteria

This systematic review and meta-analysis was designed in accordance with the International Prospective Register of Systematic Reviews (PROSPERO), registration number CRD420261329485. It followed the Preferred Reporting Items for Systematic Reviews and Meta-Analyses (PRISMA) and adhered to the participants, interventions, comparators, outcomes, and study design (PICOS) criteria shown in Table 1 [23].

### 2.2. Information Sources and Search Strategy

PubMed, Scopus, Embase (via Ovid), Web of Science, ScienceDirect, and Google Scholar were searched to identify studies assessing the anti-hyperglycemic effects of AM. Search terms were adapted for each database, with no restriction on publication date; therefore, results include studies indexed from database inception through 2 March 2026, as shown in Supplementary Tables S1–S6. Searches were restricted to English-language records.

### 2.3. Inclusion and Exclusion Criteria

The inclusion criteria of this study were as follows: (1) experimental in vivo animal studies; (2) studies with a separate control group (randomized or non-randomized); (3) studies using animal models of diabetes mellitus or otherwise exhibiting elevated blood glucose (e.g., STZ, alloxan, high-fat diet, genetic models); (4) studies administering isolated/purified AM; (5) studies that report at least one pre-specified outcome: glucose, HbA1c, insulin, or HOMA-IR; and (6) articles published as full-text in English.

Any of the following was excluded: (1) non-in vivo studies: in vitro, ex vivo, cell culture, organ bath, and computational studies; (2) studies without a separate control group (single-arm, case series, case reports); (3) animal models not exhibiting diabetes or elevated blood glucose (healthy animals without induced/metabolic hyperglycemia); (4) interventions not limited to isolated/purified AM (crude/uncharacterized Garcinia extracts, multi-compound formulations, or AM combined with other agents); (5) studies that do not report any pre-specified outcomes (glucose, HbA1c, insulin, HOMA-IR); and non-English articles (conference abstracts, posters, protocols, reviews, editorials, and other non-primary research without extractable full-text data).

### 2.4. Study Selection

After duplicate records were removed, two investigators (M.C. and A.P.) independently screened titles and abstracts against the inclusion and exclusion criteria. Study selection proceeded in two stages: independent title/abstract screening followed by independent full-text assessment, both using a standardized screening form. Interrater agreement was quantified using Cohen’s kappa (κ) with 95% confidence intervals for both screening stages. The kappa coefficient was interpreted as follows: 0–0.20 (no agreement), 0.21–0.39 (minimal agreement), 0.40–0.59 (weak agreement), 0.60–0.79 (moderate agreement), 0.80–0.90 (strong agreement), and >0.90 (almost perfect agreement) [24]. A third reviewer (F.K.) adjudicated any unresolved disagreements.

### 2.5. Data Extraction

Data were independently extracted by two researchers (M.C. and A.P.) using a standardized form. Extracted items included the first author’s last name, year of publication, study region, species and sex, diabetes/hyperglycemia model, control type, purity of AM, number of animals per treatment and control group, AM dose (mg/kg/day), route of administration, duration (weeks), outcomes for glucose, HbA1c, insulin, HOMA-IR. Discrepancies between M.C. and A.P. were resolved by a third independent researcher (F.K.), who adjudicated the disputed items. When data were unclear or missing, we contacted corresponding authors by email for clarification. Reported glucose values in mg/dL were converted to mmol/L by dividing by 18. Standard errors of the mean (SEM) were converted to standard deviations (SD) by multiplying the SEM by the square root of the corresponding sample size (SD = SEM × √n).

### 2.6. Quality Assessment

The methodological quality of included animal studies was independently evaluated using the SYRCLE risk of bias (RoB) tool, which is specifically adapted from the Cochrane RoB tool for preclinical studies [25]. The assessment covers ten domains, including selection bias (D1–D3), performance bias (D4–D5), detection bias (D6–D7), attrition bias (D8), reporting bias (D9), and other potential sources of bias (D10). Each domain was judged as “low risk” (+), “unclear risk” (−), or “high risk” (×) based on the information reported in the included studies. The evaluated domains comprised sequence generation (D1), baseline characteristics (D2), allocation concealment (D3), random housing (D4), blinding of caregivers or investigators (D5), random outcome assessment (D6), blinding of outcome assessors (D7), completeness of outcome data (D8), selective outcome reporting (D9), and other sources of bias (D10). Two reviewers independently performed the risk of bias assessment, and any discrepancies were resolved through discussion until consensus was reached.

### 2.7. Data Synthesis and Data Analysis

Efficacy of the AM treatment group compared with control for outcomes (glucose, HbA1c, and HOMA-IR) was assessed using mean differences (MD) with 95% confidence intervals (CI). For insulin, which was reported in different units across studies, standardized mean differences (SMD) were calculated using Hedges’ g. All analyses were performed in Stata version 18 (StataCorp LLC, College Station, TX, USA) and results are presented as forest plots. A random-effects model was used, and statistical significance was set at *p* < 0.05. Heterogeneity was assessed with Cochran’s Q test and quantified using *I*^2^, which was interpreted as low (0–25%), moderate (26–50%), substantial (51–75%), or considerable (>75%) [26]. Subgroup analyses were conducted to explore sources of heterogeneity by study region, species (rat vs. mouse), diabetes model (olanzapine vs. STZ vs. alloxan vs. HFD vs. genetic), dose category, route (oral vs. intraperitoneal), and treatment duration. Sensitivity analyses included leave-one-out analysis to evaluate the influence of individual studies on pooled estimates [27]. Sensitivity analyses were also performed by excluding studies that did not report the purity of AM to evaluate whether the pooled effects were robust when restricted to studies using chemically characterized (purified) AM. This analysis aimed to evaluate whether the pooled effects were robust to restriction of evidence to studies using chemically characterized (purified) AM. Publication bias was assessed using funnel plots and tested with Egger’s and Begg’s tests (*p* < 0.05 indicating potential bias) [28].

## 3. Results

### 3.1. Literature Search and Selection Procedure

**Figure 1:**
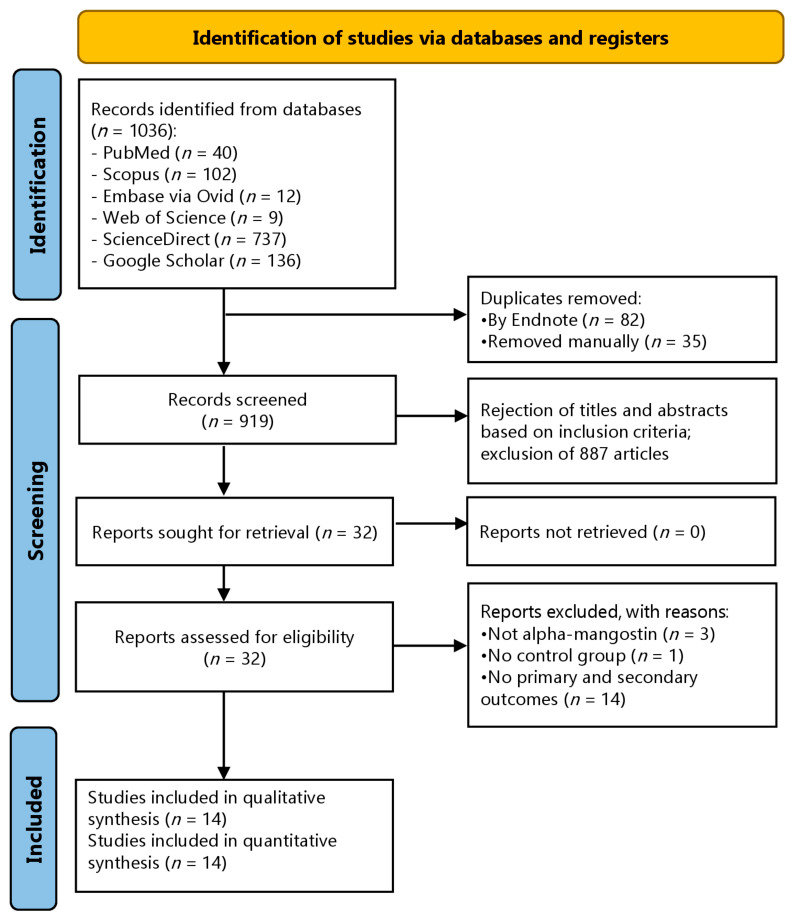
PRISMA flow chart of selected studies.

A total of 1036 records were identified from six databases: 40 from PubMed, 102 from Scopus, 12 from Embase via Ovid, 9 from Web of Science, 737 from ScienceDirect, and 136 from Google Scholar, as shown in Figure 1. After removal of 117 duplicates (82 by EndNote and 35 manually), 919 records remained and were screened by title and abstract; 887 records were excluded at this stage. Thirty-two reports were retrieved for full-text assessment, none of which were unavailable. Of these, 18 reports were excluded (not reporting isolated AM, *n* = 3; no control group, *n* = 1; did not report prespecified primary or secondary outcomes, *n* = 14), leaving 14 studies for inclusion. All 14 studies were included in both the qualitative synthesis and the quantitative meta-analysis. Interrater agreement for title–abstract screening was almost perfect agreement (Cohen’s κ = 0.94), and agreement for full-text screening was strong agreement (Cohen’s κ = 0.87), indicating high consistency between reviewers as shown in Tables S7 and S8.

### 3.2. Characteristics of Included Studies

Fourteen preclinical studies (*n* = 14; published 2013–2025) were included from Iran (*n* = 1) [20], China (*n* = 3) [17,21,22], South Korea (*n* = 2) [18,19], Thailand (*n* = 2) [29,30], India (*n* = 2) [31,32], and Indonesia (n = 4) [33,34,35,36] as shown in Table 2. Nine studies used Wistar or Sprague–Dawley rats (*n* = 9) [20,22,29,30,31,32,33,35,36] and five used mice (*n* = 5; C57BL/6, C57BL/KsJ-db/db or Mus musculus) [17,18,19,21,34]. All studies used male animals (*n* = 13) [17,18,19,21,22,29,30,31,32,33,34,35,36] except Ardakanian et al. (2022), which used female Wistar rats (*n* = 1) [20]. Disease models comprised diet- or drug-induced metabolic disorders: high-fat diet (HFD) (*n* = 2) [18,19], high-fat/high-glucose diet (HF/HG) with streptozotocin (STZ) [17,29,30,33,35,36] or alloxan administration [34], olanzapine-induced metabolic disturbances [20], and STZ-induced diabetes [31,32], and a genetic diabetic model (C57BL/KsJ-db/db) [21]. Purified AM was reported in nine studies (*n* = 9; ≥90 to ≥98% or 95–99% purity) and not reported in five studies (*n* = 5) [22,31,32,33,36]. Treatment group sizes ranged from 5 to 14 per group across the 14 studies. AM doses varied (5–200 mg/kg/day, with multi-dose regimens in several studies such as 5–20, 10–50, 25–100, 10–40, and 100–200 mg/kg/day). Administration was predominantly oral (*n* = 11) [17,18,19,29,30,31,32,33,34,35,36] with intraperitoneal injection in three studies (*n* = 3) [20,21,22]. Treatment durations ranged from 1 week to 40 weeks. Reported metabolic outcomes included fasting blood glucose/fasting blood sugar/glucose, HbA1c, serum insulin, and HOMA-IR.

### 3.3. Risk of Bias Assessment

**Figure 2:**
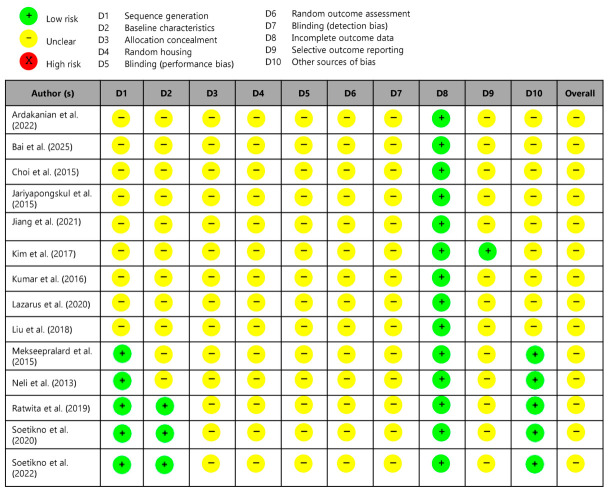
Risk of bias assessment of included animal studies (*n* = 14) using the SYRCLE tool [17,18,19,20,21,22,29,30,31,32,33,34,35,36]. Each study was evaluated across ten domains, including sequence generation (D1), baseline characteristics (D2), allocation concealment (D3), random housing (D4), blinding of caregivers or investigators (D5), random outcome assessment (D6), blinding of outcome assessors (D7), incomplete outcome data (D8), selective outcome reporting (D9), and other sources of bias (D10). The risk of bias for each domain is indicated as low risk (+, green), unclear risk (−, yellow), or high risk (×, red).

The per-study risk of bias assessment using the SYRCLE tool is presented in Figure 2. Overall, the majority of included studies demonstrated a predominance of unclear risk across most methodological domains. Specifically, domains related to allocation concealment (D3), random housing (D4), blinding of caregivers or investigators (D5), random outcome assessment (D6), blinding of outcome assessors (D7), and selective outcome reporting (D9) were consistently rated as unclear in nearly all studies, reflecting insufficient reporting of experimental procedures. In contrast, incomplete outcome data (D8) was uniformly assessed as low risk across all included studies, indicating that outcome reporting was generally complete with no evident attrition bias. Similarly, some studies demonstrated low risk in sequence generation (D1) and baseline characteristics (D2), particularly in more recent studies such as Ratwita et al. (2019) and Soetikno et al. (2020, 2022), where randomization and comparable group characteristics were explicitly reported [34,35,36]. Furthermore, several studies were rated as low risk in other sources of bias (D10), indicating consistent experimental design and appropriate use of control groups. However, no study achieved low risk across all domains, as methodological details regarding blinding and allocation concealment were largely absent. Overall, these findings indicate that while outcome reporting and experimental design were generally adequate, the overall internal validity of the included studies is limited by insufficient reporting of key methodological practices.

### 3.4. Primary Outcome

#### Effect of AM on Blood Glucose Level

**Figure 3:**
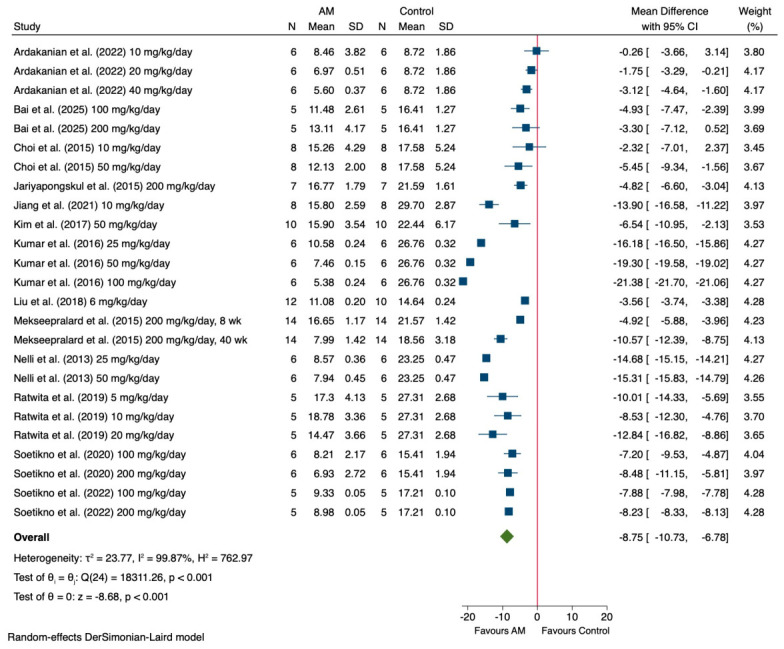
Forest plot of the effect of alpha-mangostin (AM) on blood glucose (random-effects DerSimonian–Laird model) [17,18,19,20,21,22,29,30,31,32,34,35,36]. Individual study comparisons (25 comparisons from 14 studies) are shown with mean differences and 95% confidence intervals (CI); box size indicates study weight, and horizontal lines indicate 95% CI. The green diamond represents the pooled mean difference.

A random-effects meta-analysis (DerSimonian–Laird) of 25 comparisons from 13 studies showed that treatment with AM significantly reduced blood compared with control (mean difference (MD) = −8.75 mmol/L; 95% CI: −10.73 to −6.78, *p* < 0.001), as illustrated in Figure 3 [17,18,19,20,21,22,29,30,31,32,34,35,36]. However, between-study heterogeneity was extremely high (*I*^2^ = 99.87%, *p* < 0.001), indicating considerable variation in effect sizes across studies. Overall, the pooled estimate suggests that AM is effective in lowering blood glucose, but the marked heterogeneity underscores the need for cautious interpretation and further investigation into sources of variability.

### 3.5. Secondary Outcomes

#### 3.5.1. Effect of AM on HbA1c

**Figure 4:**
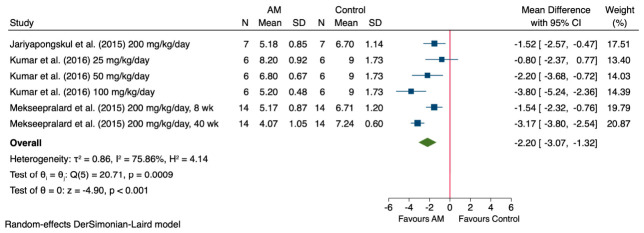
Forest plot of effect of alpha-mangostin (AM) on HbA1c levels [29,30,31]. Individual study comparisons (6 comparisons from 3 studies) are shown with mean differences and 95% confidence intervals; box size indicates study weight and horizontal lines indicate 95% CI. The diamond represents the pooled mean difference [29,30,31].

Focusing on the effect of AM on HbA1c, a random-effects meta-analysis encompassed six comparisons from three studies, as shown in Figure 4 [29,30,31]. The pooled mean difference was −2.20% (95% CI: −3.07 to −1.32; *p* < 0.001), indicating that AM consistently lowered HbA1c across studies. Individual study estimates ranged from −0.80% to −3.80%, with the majority favoring AM. Between-study heterogeneity was considerable (*I*^2^ = 75.86%, *p* = 0.0009), suggesting notable variability in effect sizes. Despite this heterogeneity, the direction of effect was consistent, and the overall findings support a beneficial impact of AM on HbA1c reduction.

#### 3.5.2. Effect of AM on Insulin Levels

**Figure 5:**
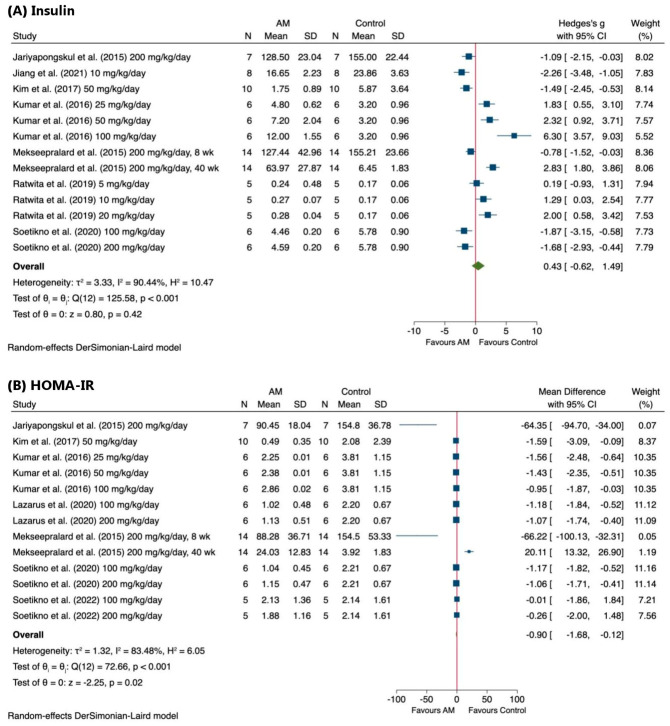
Forest plots of the effects of alpha-mangostin (AM) on (**A**) insulin (Hedges’ g) [19,21,29,30,31,34,35] and (**B**) HOMA-IR (mean difference) [19,29,30,31,33,35,36], using random-effects DerSimonian–Laird models. Box sizes indicate study weight; horizontal lines indicate 95% CIs; diamonds indicate pooled estimates.

A random-effects meta-analysis of thirteen comparisons from seven studies assessed the effect of AM on insulin levels as shown in Figure 5A. The pooled effect size, expressed as Hedges’ g, indicated no significant overall difference between AM and control groups (Hedges’ g = 0.43; 95% CI: −0.62 to 1.49, *p* = 0.42). Between-study heterogeneity was very high (*I*^2^ = 90.44%, *p* < 0.001), reflecting considerable variability in individual study estimates (some favoring AM, others favoring control). Although several studies suggested potential benefits or harms, the overall pooled estimate did not support a statistically significant effect of AM on insulin level.

#### 3.5.3. Effect of AM on Homeostatic Model Assessment of Insulin Resistance (HOMA-IR)

The data of thirteen comparisons across seven studies showed that the pooled mean difference indicated a statistically significant reduction in HOMA-IR among participants receiving AM compared with controls (MD = −0.90; 95% CI: −1.68 to −0.12, *p* = 0.02), as shown in Figure 5B. Individual study estimates varied substantially, ranging from large decreases in HOMA-IR (−64.35 to −66.22) to modest or negligible effects. Between-study heterogeneity was high (*I*^2^ = 83.48%, *p* < 0.001), indicating considerable variability across studies. Despite this heterogeneity, the direction of effect consistently favored AM, suggesting that AM supplementation may be beneficial in improving insulin resistance as measured by HOMA-IR.

### 3.6. Subgroup Analysis

**Figure 6:**
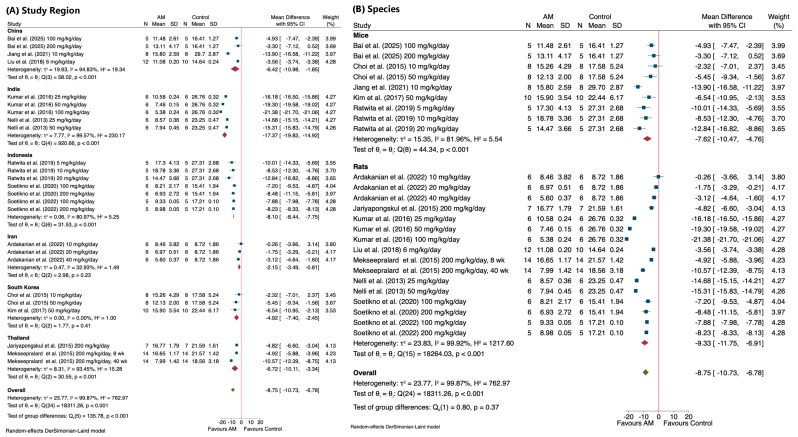
Subgroup analyses of the effect of AM on blood glucose by (**A**) study region and (**B**) animal species [17,18,19,20,21,22,29,30,31,32,34,35,36]. For each region, pooled mean differences (MDs) with 95% confidence intervals (CIs) are shown, along with heterogeneity statistics (*I*^2^). Individual study estimates are represented by squares proportional to study weight, and diamonds indicate pooled subgroup (red diamonds) and overall effects (green diamond). All analyses were conducted using random-effects models (DerSimonian–Laird).

Subgroup analysis by study region showed AM reduced blood glucose in all regions with varying magnitudes: China −6.42 mmol/L (95% CI: −10.98 to −1.85), India −17.37 mmol/L (95% CI: −19.82 to −14.92), Indonesia −8.10 mmol/L (95% CI: −8.44 to −7.75), Iran −2.15 mmol/L (95% CI: −3.49 to −0.81), South Korea −4.92 mmol/L (95% CI: −7.40 to −2.45), and Thailand −6.72 mmol/L (95% CI: −10.11 to −3.34) as shown in Figure 6A. Significant within-group *p* values for China (*I*^2^ = 94.83, *p* < 0.001), India (*I*^2^ = 99.57, *p* < 0.001), Indonesia (*I*^2^ = 80.97, *p* < 0.001), and Thailand (*I*^2^ = 93.45, *p* < 0.001) indicate considerable heterogeneity in those subgroups; non-significant *p* values for Iran (*I*^2^ = 32.93, *p* = 0.23) and South Korea (*I*^2^ = 0, *p* = 0.41) indicate more consistent effects. The test for subgroup differences was significant (*p* < 0.001), suggesting country-level factors contribute to effect variation.

Subgroup analyses by species demonstrated that AM reduced blood glucose in both mice and rats, as shown in Figure 6B. In mice (9 comparisons from 5 studies), the pooled mean difference was −7.62 mmol/L (95% CI: −10.47 to −4.76; *I*^2^ = 81.96%, *p* < 0.001), and in rats (16 comparisons from 7 studies), the pooled mean difference was −9.33 mmol/L (95% CI: −11.75 to −6.91; *I*^2^ = 99.92%, *p* < 0.001). Although the point estimate was larger in rats, the test for subgroup differences was not significant (*p* = 0.37), indicating no statistical evidence of effect modification by species.

**Figure 7:**
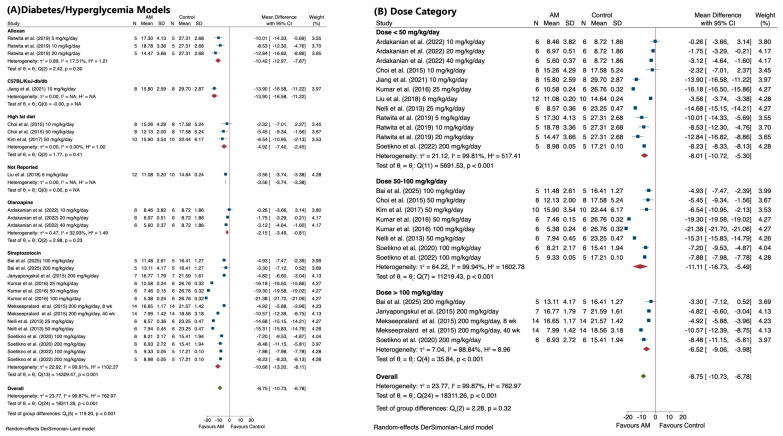
Subgroup analyses of the effect of AM on blood glucose by (**A**) diabetes/hyperglycemia models and (**B**) dose category [17,18,19,20,21,22,29,30,31,32,34,35,36]. For each region, pooled mean differences (MDs) with 95% confidence intervals (CIs) are shown, along with heterogeneity statistics (*I*^2^). Individual study estimates are represented by squares proportional to study weight, and diamonds indicate pooled subgroup (red diamonds) and overall effects (green diamond). All analyses were conducted using random-effects models (DerSimonian–Laird).

Subgroup analyses stratified by diabetes/hyperglycemia model indicated that AM reduced blood glucose across all model types, although the magnitude of the effect varied, as shown in Figure 7A. The largest pooled reduction was observed in the genetic diabetic model (C57BL/KsJ-db/db), but this estimate was based on a single study (MD = −13.90 mmol/L; 95% CI: −16.58 to −11.22). Substantial reductions were also seen in STZ-induced diabetic models (MD = −10.66 mmol/L; 95% CI: −13.20 to −8.11; *I*^2^ = 99.91%, *p* < 0.001) and in the alloxan-induced model, which was also represented by a single study (MD = −10.42 mmol/L; 95% CI: −12.97 to −7.87; *I*^2^ = 17.51%, *p* = 0.30). More modest effects were observed in high-fat diet models (MD = −4.92 mmol/L; 95% CI: −7.40 to −2.45; *I*^2^ = 0%, *p* = 0.41), olanzapine-induced metabolic disturbance models (MD = −2.15 mmol/L; 95% CI: −3.49 to −0.81; *I*^2^ = 32.93%, *p* = 0.23), and studies in which the disease model was not reported (MD = −3.56 mmol/L; 95% CI: −3.74 to −3.38). The test for subgroup differences was significant (*p* < 0.001), indicating that the type of diabetes/hyperglycemia model contributed to variability in effect size. Overall, AM appeared most effective in established diabetic models (genetic or chemically induced), but the reliance on single-study estimates for some models and high heterogeneity in several subgroups warrant cautious interpretation.

Subgroup analysis by dose category indicated that AM lowered blood glucose across all dose ranges, with point estimates as follows: <50 mg/kg/day, MD of −8.01 mmol/L (95% CI: −10.72 to −5.30; *I*^2^ = 99.81%, *p* < 0.001); 50–100 mg/kg/day, MD of −11.11 mmol/L (95% CI: −16.73 to −5.49; *I*^2^ = 99.94%, *p* < 0.001); and >100 mg/kg/day, MD of −6.52 mmol/L (95% CI −9.06 to −3.98; *I*^2^ = 88.84%, *p* < 0.001) as shown in Figure 7B. All dose subgroups favored AM, with the largest point estimate in the 50–100 mg/kg/day category, but heterogeneity was considerable within each subgroup. The test for subgroup differences (*p* = 0.32) indicates no statistically significant effect modification by dose category. These findings suggest a consistent glucose-lowering effect of AM across doses, though the substantial within-group heterogeneity warrants cautious interpretation and further dose–response investigation.

**Figure 8:**
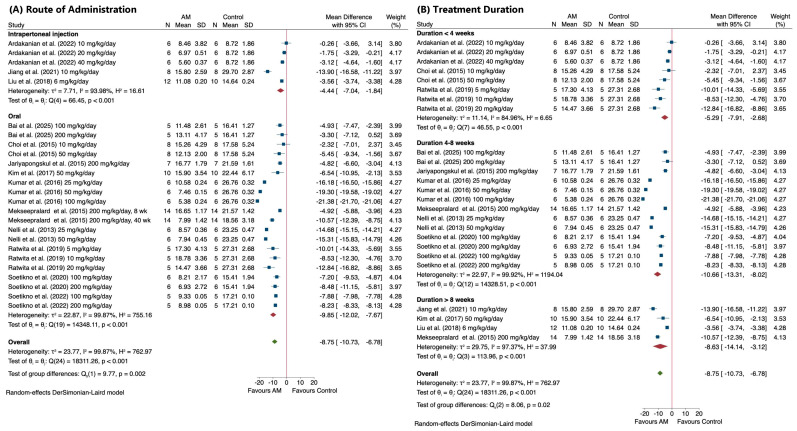
Subgroup analyses of the effect of AM on blood glucose by (**A**) route of administration and (**B**) treatment duration [17,18,19,20,21,22,29,30,31,32,34,35,36]. For each region, pooled mean differences (MDs) with 95% confidence intervals (CIs) are shown, along with heterogeneity statistics (*I*^2^). Individual study estimates are represented by squares proportional to study weight, and diamonds indicate pooled subgroup (red diamonds) and overall effects (green diamond). All analyses were conducted using random-effects models (DerSimonian–Laird).

Subgroup analysis by route of administration showed that AM reduced blood glucose through both intraperitoneal and oral delivery, although the magnitude of reduction differed between the two routes, as shown in Figure 8A. Intraperitoneal administration produced a pooled MD of −4.44 mmol/L (95% CI: −7.04 to −1.84) with considerable heterogeneity (*I*^2^ = 93.98%, *p* < 0.001). In contrast, oral administration resulted in a much larger pooled reduction of −9.85 mmol/L (95% CI: −12.02 to −7.67), accompanied by considerable heterogeneity (*I*^2^ = 99.87%, *p* < 0.001). The test for subgroup differences was statistically significant (*p* = 0.002), suggesting that the route of administration may influence the glucose-lowering effect of AM. Overall, while AM lowered glucose regardless of the administration route, oral delivery appeared to produce a greater effect; however, the consistently high heterogeneity across subgroups suggests the need for careful interpretation.

Subgroup analysis by treatment duration showed that AM reduced blood glucose across all treatment durations, although the magnitude of reduction varied, as shown in Figure 8B. Studies with short-term treatment (<4 weeks) demonstrated a pooled MD of −5.29 mmol/L (95% CI: −7.91 to −2.68), with considerable heterogeneity (*I*^2^ = 84.96%, *p* < 0.001). In the 4–8-week subgroup, the glucose-lowering effect was considerably larger, yielding a pooled mean difference of −10.66 mmol/L (95% CI: −13.31 to −8.02), again with considerable heterogeneity *(I*^2^ = 99.92%, *p* < 0.001). Studies with longer treatment durations (>8 weeks) produced a pooled mean difference of −8.63 mmol/L (95% CI: −14.14 to −3.12), with moderate heterogeneity (*I*^2^ = 37.99%, *p* < 0.001). The test for subgroup differences was statistically significant (*p* = 0.02), suggesting that treatment duration may influence the magnitude of AM’s glucose-lowering effect. Overall, AM demonstrated glucose-lowering benefits across all durations, with the strongest effect observed in the 4–8-week range; however, the considerable heterogeneity across subgroups suggests the need for careful interpretation.

### 3.7. Sensitivity Analysis

**Figure 9:**
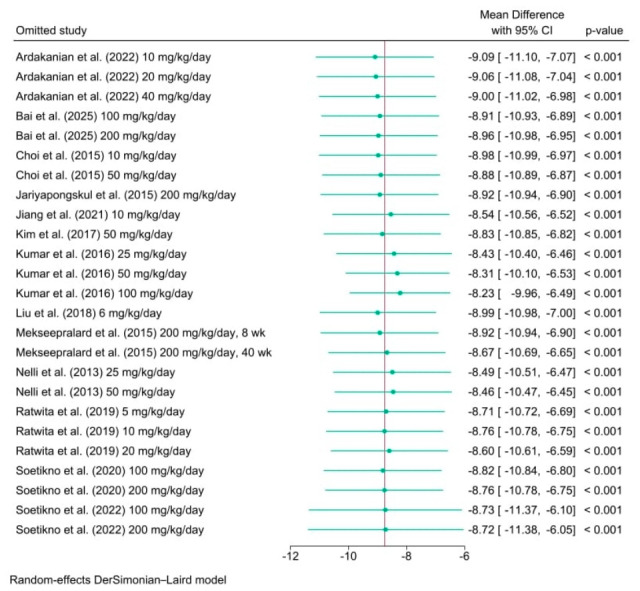
Leave-one-out sensitivity analysis for the effect of AM on blood glucose (random-effects DerSimonian–Laird model) [17,18,19,20,21,22,29,30,31,32,34,35,36]. Each row shows the pooled mean difference (MD) and 95% CI recalculated after omitting the indicate study. Green dots represent the pooled MD estimates, and horizontal lines indicate their corresponding 95% confidence intervals. The vertical line denotes the overall MD from the primary analysis.

A leave-one-out sensitivity analysis using a random-effects DerSimonian–Laird model demonstrated that the pooled effect of AM on blood glucose was robust to the exclusion of any single study, as shown in Figure 9. Omitting each study in turn yielded pooled MDs ranging from −9.09 mmol/L to −8.23 mmol/L, with all iterations remaining statistically significant at *p* < 0.001. The narrow spread of estimates and consistent significance indicate that no individual study influenced the overall pooled effect, supporting the stability of the glucose-lowering association.

A sensitivity analysis was conducted, excluding five studies in which the purity of AM was not reported. This analysis included eight studies comprising 15 comparisons, all of which used chemically characterized (purified) AM. Treatment with AM remained associated with a statistically significant reduction in blood glucose compared with control groups (MD of −6.15 mmol/L; 95% CI: −8.01 to −4.29; *p* < 0.001) as shown in Supplementary Figure S1.

### 3.8. Publication Bias

**Figure 10:**
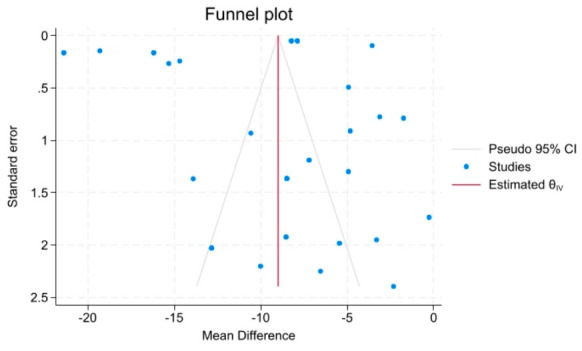
Funnel plot for assessment of publication bias for the effect of AM on blood glucose (mean difference or MD) [17,18,19,20,21,22,29,30,31,32,34,35,36]. Each point represents an individual study; the vertical red line indicates the pooled MD, and the grey lines indicate the pseudo 95% CI.

Assessment of publication bias using visual inspection of the funnel plot revealed asymmetry, suggesting the possibility of small-study effects as shown in Figure 10. This visual impression was supported by Egger’s regression test, which indicated statistically significant asymmetry (*p* = 0.0247), consistent with potential publication bias. In contrast, Begg’s rank correlation test did not detect significant asymmetry (*p* = 0.5910), suggesting that the evidence for publication bias was not consistent across methods (region, species, model, dose, route and duration). Overall, while Egger’s test points to potential small-study or selective reporting effects, the nonsignificant Begg’s test and the distribution of studies in the funnel plot imply that any publication bias, if present, is likely modest and should be interpreted with caution.

## 4. Discussion

Type 2 diabetes mellitus is characterized by chronic hyperglycemia, reflected by elevated fasting blood glucose (FBG), increased HbA1c, and dysregulated insulin levels with higher HOMA-IR, all of which are associated with impairments in β-cell mass and function [37,38]. This systematic review and meta-analysis synthesized evidence from 14 preclinical studies to evaluate the anti-hyperglycemic effects of AM across diverse animal models of diabetes and metabolic dysfunction. The pooled estimate from 25 comparisons showed a significant reduction in fasting blood glucose, accompanied by reductions in HbA1c and HOMA-IR, while effects on circulating insulin were heterogeneous and not statistically significant. The greatest glucose-lowering effects were observed in chemically or genetically induced diabetic models (STZ, alloxan, db/db), with more modest effects in diet-induced or drug-induced metabolic disturbance models, and variability according to dose, route of administration, and treatment duration. Although most included studies were rated as unclear risk due to insufficient reporting of methodological practices, sensitivity analyses showed that the pooled results were robust. Excluding four studies with unreported AM purity produced statistically significant and directionally consistent effects, indicating that the glucose-lowering association was not driven by compound characterization. However, substantial heterogeneity and evidence of small-study effects (Egger’s test) warrant cautious interpretation and highlight the need for well-designed, adequately powered preclinical studies.

AM produced a substantial pooled reduction in blood glucose in our meta-analysis, suggesting a consistent antihyperglycemic effect across heterogeneous preclinical settings, although this finding should be interpreted with caution due to considerable between-study heterogeneity. Mechanistically, AM has been shown to activate AMPK/SIRT1 signaling, increase PPARγ expression, and upregulate GLUT transporters, which together suppress hepatic gluconeogenesis and enhance skeletal-muscle glucose uptake, suggesting potential mechanisms contributing to glucose lowering without requiring increased insulin secretion [19,20]. The proposed mechanisms of AM should be interpreted cautiously, as they are primarily derived from preclinical evidence and may involve multiple interacting signaling pathways. AM also attenuates oxidative and inflammatory stress via Nrf2/HO-1 induction and NF-κB inhibition, mechanisms closely linked to hepatic and peripheral insulin sensitivity and consequent reductions in circulating glucose [18,26]. In diabetic cardiometabolic models, AM reduces lipotoxicity and oxidative damage through the AKT/FOXO1/CD36 signaling pathway and bolsters endogenous antioxidant programs (Nrf2, HO-1, SOD2), mechanistically linking decreased lipid influx/ROS to improved insulin action and glycemic control [17]. However, considerable heterogeneity likely reflects variation in species, dose, route, and duration; these methodological differences complicate precise effect estimation and warrant standardized preclinical protocols.

The pooled HbA1c reduction indicates sustained glycemic improvement rather than only transient effects on fasting blood glucose. Mechanisms that plausibly underlie chronic glycemic benefits include long-term preservation of β-cell mass, attenuation of glucotoxicity, and decreased systemic inflammation and oxidative stress, pathways that have been demonstrated for AM and other plant polyphenols in animal studies [29,30,39,40]. This aligns with a preclinical report in type 2 diabetic rats showing that both 8 and 40 weeks of oral AM (200 mg/kg/day) lowered HbA1c together with fasting glucose and lipids, with the longer regimen also improving insulin profiles and glucose tolerance [30]. Consistent findings were observed in another STZ/HFD rat model, in which AM (200 mg/kg/day, 8 weeks) reduced HbA1c alongside oxidative/inflammatory retinal biomarkers, reinforcing the link between improved tissue redox state and chronic glycemic indices [29]. Although human data remain limited, administration of *Garcinia mangostana* L. extract has been associated with improvements in endothelial dysfunction accompanied by reductions in fasting blood glucose and HbA1c in subjects with type 2 diabetes and high Framingham risk scores [41]. Although derived from fewer comparisons than the glucose analysis, the consistent direction of effect across studies suggests that AM’s integrated metabolic and cytoprotective mechanisms may contribute to improvements in chronic glycemic indices; however, their clinical relevance remains to be established.

AM demonstrated inconsistent changes in circulating insulin yet a statistically significant improvement in HOMA-IR, suggesting that its primary action may be to enhance insulin sensitivity rather than increase insulin secretion. In our pooled analyses, insulin concentrations were heterogeneous and nonsignificant (Hedges’ g = 0.43), with individual studies reporting increases, decreases, or no change [19,21,29,30,31,34,35]. By contrast, HOMA-IR was reduced across studies that reported it [19,29,30,31,33,35,36], supporting improved insulin sensitivity. However, HOMA-IR was originally developed for assessing insulin resistance in humans, its application in rodent models should be interpreted cautiously and considered primarily as a relative index for within-study comparison rather than an absolute metabolic measure. The observed reduction in HOMA-IR is nevertheless consistent with tissue-level actions of AM, including AMPK activation, improved Akt signaling, increased GLUT4 translocation, and reduced NF-κB–mediated inflammation that enhance insulin-signaling efficiency at both hepatic and peripheral sites [17,19,20]. These insulin-sensitizing actions are further supported by reductions in tissue oxidative stress markers (MDA, AGEs, RAGE, TNF-α) reported in diabetic rat models [29]. Importantly, early human evidence points in the same direction: a randomized controlled pilot trial in obese insulin-resistant women demonstrated a 53.22% reduction in HOMA-IR after 26 weeks of mangosteen extract supplementation, despite fasting insulin not being significantly elevated, indicating enhanced insulin sensitivity rather than increased insulin secretion [42]. Taken together, these findings suggest that AM’s metabolic benefits arise primarily from improved insulin action, achieved through coordinated modulation of cellular energy pathways, redox balance, and lipid handling, while changes in absolute insulin levels depend on underlying β-cell status and disease progression.

Subgroup analyses showed context-dependent variation in AM’s glucose-lowering magnitude. By region, effect sizes differed across countries (China, India, Indonesia, Iran, South Korea, Thailand), likely reflecting variation in animal strains, diets, laboratory practices, and AM purity. By species, both mice and rats demonstrated glucose reductions with similar directionality, although point estimates were larger in rats without a significant species interaction. By disease model, the largest effects occurred in genetically or chemically induced diabetes (db/db, STZ, alloxan), while diet-induced and olanzapine models showed more modest reductions, which may suggest greater efficacy in models characterized by β-cell dysfunction or severe hyperglycemia, although this observation should be interpreted cautiously. By dose category, all ranges (<50, 50–100, >100 mg/kg/day) favored AM, with the 50–100 mg/kg group showing the largest point estimate, but no clear dose–response was demonstrated. By route, oral administration produced a larger pooled glucose reduction than intraperitoneal dosing, which may reflect differences in absorption, first-pass metabolism, or exposure. By treatment duration, benefits were observed across short (<4 weeks), intermediate (4–8 weeks), and long (>8 weeks) regimens, with the 4–8-week interventions showing the greatest point estimate. Across the included preclinical studies, AM administration ranging from short-term (1 week) to long-term exposure (40 weeks) was not associated with reported treatment-related toxicity, although systematic safety and toxicological assessments were not consistently performed and therefore definitive conclusions on long-term safety cannot be drawn. However, considerable heterogeneity persisted within most subgroups, several subgroup estimates were based on few or single studies, and variability in administration route, dose, treatment duration, and metabolic handling limits precise quantitative inference; therefore, standardized, adequately powered preclinical designs incorporating pharmacokinetic and metabolic profiling are needed to identify true effect modifiers and clarify key bioactive substrates.

Title screening and full-text selection demonstrated high reviewer agreement, supporting reliable study identification. Risk-of-bias assessment using the SYRCLE tool indicated that most included studies were rated as unclear risk due to insufficient reporting of key methodological practices, particularly randomization and blinding. Leave-one-out sensitivity analyses showed the pooled glucose effect was robust to the exclusion of any single study, indicating no single study unduly influenced the main estimate. In addition, sensitivity analyses excluding studies with unreported AM purity yielded a statistically significant and directionally consistent pooled effect, indicating that the observed glucose-lowering association was not driven by uncertainty in compound characterization. Assessment of small-study effects produced mixed findings: visual funnel-plot asymmetry and a significant Egger’s test suggest possible publication or small-study bias, whereas Begg’s test was not significant; thus, selective reporting may be present but is likely modest. Together, these appraisals increase confidence in the observed signal while underscoring the need for larger, transparent, and preregistered preclinical studies to mitigate residual bias and improve precision.

This study has several limitations that should be considered when interpreting the findings. First, although we identified consistent metabolic benefits of AM across preclinical models, the overall evidence base remains small and heterogeneous, with several subgroup estimates derived from only a few studies or from single-study strata. Although the pooled results showed consistent effects, the relatively small number of included studies (*n* = 14) limits the overall strength of evidence and highlights the need for additional well-designed, adequately powered preclinical studies to confirm these findings. Second, most included studies had small group sizes and were conducted in male rodents, reducing generalizability across sexes. Third, heterogeneity in AM characterization (purity reported in nine studies, not reported in five studies) and formulation, as well as differences in vehicle and co-treatments, complicate dose–response interpretation. Additionally, variability in dose and treatment duration across studies may have contributed to the observed heterogeneity, limiting direct comparability and complicating interpretation of dose–response relationships. Given that AM belongs to a class of structurally related xanthone derivatives, including β- and γ-mangostin, future studies should incorporate comparative evaluations with these analogues and provide detailed characterization of molecular structure and compound purity to better elucidate structure–activity relationships and confirm the specificity of the observed biological effects. Furthermore, differences in the route of administration may influence the observed effects of AM, as oral delivery involves metabolic transformation into potentially active or inactive metabolites, whereas intraperitoneal administration may more directly reflect the activity of the parent compound, thereby contributing to variability in efficacy. Future studies should therefore systematically evaluate the impact of administration routes by integrating pharmacokinetic, metabolic, and bioavailability analyses, including identification and characterization of active metabolites, to better clarify their contribution to the observed biological effects.

This systematic review and meta-analysis provides preclinical evidence that AM exerts significant glucose-lowering and insulin-sensitizing effects across diverse animal models of metabolic dysfunction. The consistency in effect direction across studies and robustness in sensitivity analyses support the reliability of these findings, despite most studies being rated as unclear risk of bias due to insufficient reporting of methodological practices. Nevertheless, considerable heterogeneity and variability in experimental designs highlight the need for standardized, mechanistically informed, and clinically oriented research to advance AM toward translational application. However, the clinical effectiveness of AM remains uncertain, particularly in patients with complex or chronic comorbid conditions, as the current evidence is limited to preclinical models and requires validation in well-designed clinical studies.

## 5. Conclusions

Overall, this systematic review and meta-analysis suggest that AM is associated with glucose-lowering and insulin-sensitizing effects across a range of preclinical models of diabetes and metabolic dysfunction. Pooled analyses indicated reductions in fasting glucose, HbA1c, and HOMA-IR, while effects on circulating insulin were variable, collectively supporting a mechanism predominantly related to improved insulin sensitivity rather than enhanced insulin secretion. However, the magnitude of effect varied substantially according to disease model, dose, route of administration, treatment duration, and study region. Importantly, high between-study heterogeneity, limited representation of female animals, inconsistent reporting of compound characterization, and variability in experimental design preclude definitive conclusions regarding efficacy or dose optimization. Therefore, the present findings should be interpreted as indicative rather than confirmatory, highlighting the potential of AM while underscoring the need for standardized, adequately powered preclinical studies incorporating pharmacokinetics, metabolic profiling, sex-specific analyses, and safety assessments prior to progression toward clinical evaluation.

## Figures and Tables

**Table 1:** Participants, intervention, comparison, outcomes, and study design (PICOS) criteria.

Parameter	Description
Population (P)	Animal models of diabetes mellitus or animals exhibiting elevated blood glucose.
Intervention (I)	Isolated/purified alpha-mangostin administered alone (any dose, route, or duration)
Comparator (C)	Vehicle-treated, placebo-treated, or no-treatment control groups.
Outcomes (O)	Primary outcome: blood glucose level. Secondary outcomes: glycated hemoglobin (HbA1c), insulin, homeostatic model assessment of insulin resistance (HOMA-IR).
Study design (S)	Controlled in vivo experimental studies.

**Table 2:** Characteristics of the included studies.

Study ID	Author (s)	Region	Species/Sex	Model	Control Type	Type of AM	Number (Treatment/ Control)	AM’s Dose (mg/kg/day)	Route	Duration (Week)	Outcome (s)
1	Ardakanian et al., 2022 [20]	Iran	Wistar rats/female	Olanzapine-induced metabolic disorders	Vehicle	Purified AM (>90%)	(6/6)	10, 20, 40	Intraperitoneal injection	2	Primary outcome: glucose
2	Bai et al., 2025 [17]	China	C57BL/6J wild-type mice/male	HFD- and STZ-induced type 2 diabetes mellitus	Vehicle	Purified AM (≥98%)	(5/5)	100, 200	Oral	6	Primary outcome: glucose
3	Choi et al., 2015 [18]	South Korea	C57BL/6 mice/male	HFD-induced hepatic steatosis and obesity	Vehicle	Purified AM (>98%)	(8/8)	10, 50	Oral	1	Primary outcome: glucose
4	Jariyapongskul et al., 2015 [29]	Thailand	Sprague-Dawley rats/male	HFD- and STZ-induced type 2 diabetes mellitus	Vehicle	Purified AM (>95%)	(7/7)	200	Oral	8	Primary outcome: glucose; secondary outcomes: HbA1c, serum insulin, HOMA-IR
5	Jiang et al., 2021 [21]	China	C57BL/KsJ-diabetic (db/db) mice/male	Type 2 diabetes mellitus (genetic model)	Vehicle	Purified AM (≥98%)	(8/8)	10	Intraperitoneal injection	12	Primary outcome: glucose; secondary outcome: insulin
6	Kim et al., 2017 [19]	South Korea	C57BL/6 mice/male	HFD-induced obesity	Vehicle	Purified AM (95–99%)	(10/10)	50	Oral	12	Primary outcome: glucose; secondary outcome: insulin
7	Kumar et al., 2016 [31]	India	Wistar rats/male	STZ-induced type 2 diabetes mellitus	Vehicle	NR	(6/6)	25, 50, 100	Oral	8	Primary outcome: glucose; secondary outcomes: HbA1c, serum insulin, HOMA-IR
8	Lazarus et al., 2020 [33]	Indonesia	Wistar rats/male	HF/HG diet- and STZ-induced insulin resistance	Vehicle	NR	(6/6)	100, 200	Oral	8	Secondary outcome: HOMA-IR
9	Liu et al., 2018 [22]	China	Sprague Dawley rats/male	Diabetic nephropathy	Vehicle (normal saline)	NR	(12/10)	6	Intraperitoneal injection	12	Primary outcome: glucose
10	Mekseepralard et al., 2015 [30]	Thailand	Sprague-Dawley rats/male	HFD- and STZ-induced type 2 diabetes mellitus	Vehicle	Purified AM (>95%)	(14/14)	200	Oral	8, 40	Primary outcome: glucose; secondary outcomes: HbA1c, serum insulin, HOMA-IR
11	Nelli et al., 2013 [32]	India	Wistar rats/male	STZ-induced type 2 diabetes mellitus	Vehicle	NR	(6/6)	25, 50	oral	7	Primary outcome: glucose
12	Ratwita et al., 2019 [34]	Indonesia	Mus musculus mice/male	Alloxan-induced diabetes mellitus	Vehicle	Purified AM (≥98%)	(5/5)	5, 10, 20	oral	3	Primary outcome: glucose; secondary outcomes: insulin
13	Soetikno et al., 2020 [35]	Indonesia	Wistar rats/male	HF/HG diet- and STZ-induced type 2 diabetes mellitus	Vehicle	Purified AM (≥98%)	(6/6)	100, 200	oral	8	Primary outcome: glucose; secondary outcomes: insulin, HOMA-IR
14	Soetikno et al., 2022 [36]	Indonesia	Wistar rats/male	HF/HG diet- and STZ-induced type 2 diabetes mellitus	Vehicle	NR	(5/5)	100, 200	oral	8	Primary outcome: glucose; secondary outcomes: HOMA-IR

AM, alpha-mangostin; NR, not reported; HbA1c, glycated hemoglobin; HOMA-IR, homeostatic model assessment of insulin resistance.

## Data Availability

No new data were created or analyzed in this study.

## Supplementary Materials

The following supporting information can be downloaded at https://www.mdpi.com/article/10.3390/life16060906/s1. Table S1: Literature search in PubMed; Table S2: Literature search in Scopus; Table S3: Literature search in Embase (via Ovid); Table S4: Literature search in ScienceDirect; Table S5: Literature search in Web of Science; Table S6: Literature search in Google Scholar; Table S7: Inter-rater agreement for the title–abstract screening stage; Table S8: Inter-rater agreement for the full-text screening stage; Figure S1: Forest plot showing the sensitivity analysis of the effect of alpha-mangostin (AM) on blood glucose after exclusion of studies with unreported compound purity Mean differences (MDs) with 95% confidence intervals (CIs) compare AM-treated and control groups across 15 comparisons from eight studies using chemically characterised AM. The pooled estimate was calculated using a random-effects DerSimonian–Laird model. Square sizes represent study weights, horizontal lines indicate 95% CIs, and the diamond represents the overall pooled effect.

## Abbreviations

The following abbreviations are used in this manuscript:

AM	Alpha-mangostin
ACC	acetyl-CoA carboxylase
ADA	protein kinase B
AMPK	5′-adenosine monophosphate–activated protein kinase
aSMase	Acid sphingomyelinase
CD36	Cluster of differentiation 36
CI	Confidence interval
DPP-4	Dipeptidyl-peptidase-4
FBG	Fasting blood glucose
FAS	fatty acid synthase
FOXO1	Forkhead box protein O1
GLP-1	Glucagon-like peptide-1
GLUT	Glucose transporter
HbA1c	Glycated hemoglobin
HF/HG	High-fat/high-glucose
HFD	High-fat diet
HO-1	Heme oxygenase-1
HOMA-IR	Homeostatic model assessment of insulin resistance
MD	Mean difference
NF-κB	Nuclear factor kappa-light-chain-enhancer of activated B cells
NR	Not reported
Nrf2	Nuclear factor erythroid 2–related factor 2
PPARγ	Peroxisome proliferator-activated receptor gamma
PRISMA	Preferred Reporting Items for Systematic Reviews and Meta-Analyses
ROB	Risk of Bias tool
ROS	Reactive oxygen species
SD	Standard deviation
SEM	Standard error of the mean
SGLT2	Sodium–glucose cotransporter-2
SIRT1	Sirtuin 1
SMD	Standardized mean difference
SREBP-1	Sterol regulatory element-binding protein 1
STZ	Streptozotocin
T2DM	Type 2 diabetes mellitus
TZDs	Thiazolidinediones
