# Supplementary material for: Anti-Hyperglycemic Effects of Alpha-Mangostin in Animal Models: A Systematic Review and Meta-Analysis
Source: Life (Basel). 2026 May 28;16(6):906. doi: 10.3390/life16060906 (PMC13302603; doi:10.3390/life16060906)
Supplement: Supplementary file 1 [file life-16-00906-s001.zip › life-4306944-supplementary.pdf]

Anti-Hyperglycemic Effects of Alpha-Mangostin in Animal Models: A Systematic Review and Meta-Analysis

Moragot Chatatikun [1,2], Fumitaka Kawakami [3,4,5], Motoki Imai [5,6,7], Ratana Netphakdee [1], Aman Tedasen [1,2], Jongkonnee Thanasai [8], Wiyada Kwanhian Klangbud [9], Atthaphong Phongphithakchai [10,*]

[1] School of Allied Health Sciences, Walailak University, Nakhon Si Thammarat 80160, Thailand; moragot.ch@wu.ac.th; ratana.ne@mail.wu.ac.th; aman.te@wu.ac.th
[2] Research Excellence Center for Innovation and Health Products (RECIHP), Walailak University, Nakhon Si Thammarat 80160, Thailand
[3] Department of Regulation Biochemistry, Graduate School of Medical Sciences, Kitasato University, Sagamihara 252-0373, Japan; kawakami@kitasato-u.ac.jp
[4] Department of Health Administration, School of Allied Health Sciences, Kitasato University, Sagamihara 252-0373, Japan
[5] Regenerative Medicine and Cell Design Research Facility, School of Allied Health Science, Kitasato University, Sagamihara 252-0373, Japan; imai-m@kitasato-u.ac.jp
[6] Department of Molecular Diagnostics, School of Allied Health Sciences, Kitasato University, Sagamihara 252-0373, Japan
[7] Department of Applied Tumor Pathology, Graduate School of Medical Sciences, Kitasato University, Sagamihara 252-0373, Japan
[8] Faculty of Medicine, Mahasarakham University, Mahasarakham 44000, Thailand; jongkonnee@msu.ac.th
[9] Medical Technology Program, Faculty of Science, Nakhon Phanom University, Nakhon Phanom 48000, Thailand
[10] Nephrology Unit, Division of Internal Medicine, Faculty of Medicine, Prince of Songkla University, Songkhla 90110, Thailand
* Correspondence: atthaphong.p@psu.ac.th

Supplementary Table S1. Literature search in PubMed.

Search number	Search Details	Results
1	"alpha-mangostin"[Title/Abstract]	725
2	"alpha-mangostin"[Title/Abstract]	725
3	"mangostin"[Title/Abstract]	851
4	"alpha-mangostin"[Title/Abstract] OR "alpha-mangostin"[Title/Abstract] OR "mangostin"[Title/Abstract]	851
5	"diabetes mellitus"[Title/Abstract]	306,654
6	"glucose"[Title/Abstract]	636,577
7	"blood glucose"[Title/Abstract]	102,718
8	"hyperglycemia"[Title/Abstract]	63,386
9	"diabetes mellitus"[Title/Abstract] OR "glucose"[Title/Abstract] OR "blood glucose"[Title/Abstract] OR "hyperglycemia"[Title/Abstract]	882,949
10	("alpha-mangostin"[Title/Abstract] OR "alpha-mangostin"[Title/Abstract] OR "mangostin"[Title/Abstract]) AND ("diabetes mellitus"[Title/Abstract] OR "glucose"[Title/Abstract] OR "blood glucose"[Title/Abstract] OR "hyperglycemia"[Title/Abstract])	40

Supplementary Table S2. Literature search in Scopus.

Search number	Search Details	Results
1	TITLE-ABS-KEY (alpha-mangostin)	1,259
2	TITLE-ABS-KEY (α-mangostin)	1,259
3	TITLE-ABS-KEY (mangostin)	1,560
4	(TITLE-ABS-KEY (alpha-mangostin)) OR (TITLE-ABS-KEY (mangostin)) OR (TITLE-ABS-KEY (α-mangostin))	1,560
5	TITLE-ABS-KEY (diabetes mellitus)	1,119,245
6	TITLE-ABS-KEY (glucose)	1,233,433
7	TITLE-ABS-KEY (blood glucose)	610,571
8	TITLE-ABS-KEY (hyperglycemia)	154,728
9	(TITLE-ABS-KEY (diabetes mellitus)) OR (TITLE-ABS-KEY (glucose)) OR (TITLE-ABS-KEY (blood glucose)) OR (TITLE-ABS-KEY (hyperglycemia))	2,034,330
10	((TITLE-ABS-KEY (diabetes mellitus)) OR (TITLE-ABS-KEY (glucose)) OR (TITLE-ABS-KEY (blood glucose)) OR (TITLE-ABS-KEY (hyperglycemia))) AND ((TITLE-ABS-KEY (alpha-mangostin)) OR (TITLE-ABS-KEY (mangostin)) OR (TITLE-ABS-KEY (α-mangostin)))	102

Supplementary Table S3. Literature search in Embase (via Ovid).

Search number	Search Details	Results
1	alpha-mangostin.m_titl.	547
2	mangostin.m_titl.	658
3	1 or 2	658
4	diabetes mellitus.m_titl.	135,043
5	glucose.m_titl.	185,872
6	blood glucose.m_titl.	16,910
7	hyperglycemia.m_titl.	15,663
8	4 or 5 or 6 or 7	328,038
9	3 and 8	12

Supplementary Table S4. Literature search in ScienceDirect.

Search Details	Results
("alpha-mangostin" OR "α-mangostin" OR "mangostin") AND ("diabetes mellitus OR OR "glucose" OR "blood glucose" OR "hyperglycemia")	737

Supplementary Table S5. Literature search in Web of Science.

Search Details	Results
diabetes mellitus (Title) or glucose (Title) or blood glucose (Title) or hyperglycemia (Title)	243,542
alpha-mangostin (Title) or α-mangostin (Title) or mangostin (Title)	681
alpha-mangostin or α-mangostin or mangostin (Title) and diabetes mellitus or glucose or blood glucose or hyperglycemia (Title)	9

Supplementary Table S6. Literature search in Google Scholar.

Search Details	Results
(alpha-mangostin or α-mangostin or mangostin) and (diabetes mellitus or glucose or blood glucose or hyperglycemia)	136

Supplementary Table S7. Inter-rater agreement for the title–abstract screening stage.

Cohen's Kappa (κ = 0.94)	**Rater 2 (A.P.): Exclude**	**Rater 2 (A.P.): Include**	**Total Rater 1 (M.C.)**
**Rate 1 (M.C.) : Exclude**	880	0	880
**Rate 1 (M.C.) : Include**	4	35	39
**Total Rater 2 (A.P.)**	884	35	919 (Over all)

Supplementary Table S8. Inter-rater agreement for the full-text screening stage.

Cohen's Kappa (κ = 0.87)	**Rater 2 (A.P.): Exclude**	**Rater 2 (A.P.): Include**	**Total Rater 1 (M.C.)**
**Rate 1 (M.C.) : Exclude**	12	2	13
**Rate 1 (M.C.) : Include**	0	18	19
**Total Rater 2 (A.P.)**	12	20	32 (Over all)

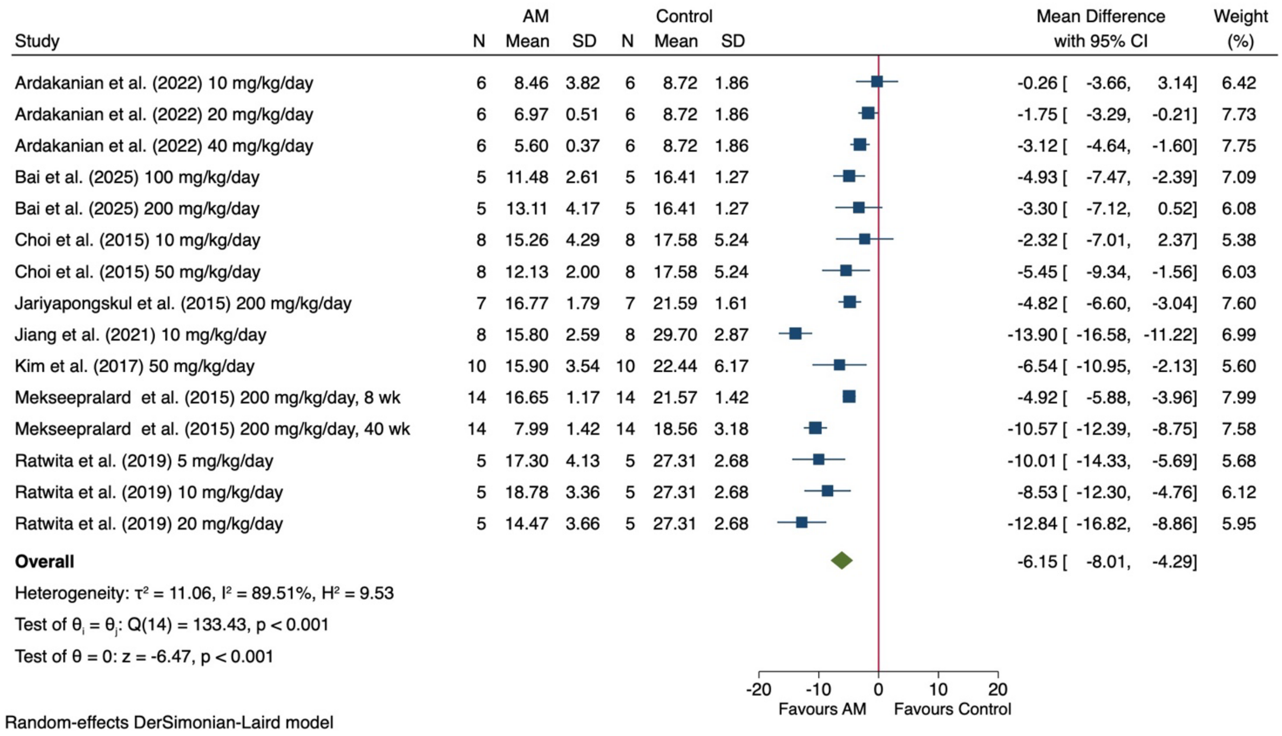

Figure S1. Forest plot showing the sensitivity analysis of the effect of alpha-mangostin (AM) on blood glucose after exclusion of studies with unreported compound purity. Mean differences (MDs) with 95% confidence intervals (CIs) compare AM-treated and control groups across 15 comparisons from eight studies using chemically characterised AM. The pooled estimate was calculated using a random-effects DerSimonian–Laird model. Square sizes represent study weights, horizontal lines indicate 95% CIs, and the diamond represents the overall pooled effect.
